# Targeted sequencing analysis of *Mycoplasma gallisepticum* isolates in chicken layer and breeder flocks in Thailand

**DOI:** 10.1038/s41598-022-14066-4

**Published:** 2022-06-14

**Authors:** Arithat Limsatanun, Somsak Pakpinyo, Kriengwich Limpavithayakul, Teerarat Prasertsee

**Affiliations:** 1 Faculty of Veterinary Science, Prince of Songkla University, Hat Yai, Kho Hong, Songkhla, 90110 Thailand; 2 Avian Health Research Unit, Department of Veterinary Medicine, Faculty of Veterinary Science, Chulalongkorn University, Patumwan, 10330 Bangkok Thailand

**Keywords:** Biological techniques, Microbiology, Diseases

## Abstract

*Mycoplasma gallisepticum* (MG) is one of the most economically important pathogens worldwide. MG affects the respiratory system and impairs growth performance in poultry. In developing countries, the most widely used technique to identify MG is the conventional PCR assay. In this study, 24 MG isolates collected from Thailand farms with unvaccinated chickens during 2002–2020 were characterized by gene-targeted sequencing (GTS), followed by phylogenetic analysis using unweighted pair group method with arithmetic mean. These 24 Thai MG isolates differed from vaccine strains, including the F, ts-11 and 6/85 strains. One isolate showed 99.5–100% genetic similarity to the F strain with 4 partial gene analyses. This result may have been due to contamination from vaccinated flocks because the F strain is the most commonly used vaccine strain in Thailand. However, the GTS analysis using the partial MG genes in this study showed that the isolates could be grouped into different patterns based on individual gene sequences. The phylogenetic analysis of partial *mgc2*, *gapA*, *pvpA* and *lp* gene sequences classified the Thai MG isolates into 7, 11, 7 and 2 groups, respectively. In conclusion, at least 2 partial MG genes, especially partial *gap*A and *mgc*2 genes, are needed to differentiate MG isolates.

## Introduction

*Mycoplasma gallisepticum* (MG) remains one of the most important bacterial pathogens worldwide, causing a respiratory disease called chronic respiratory disease (CRD) in infected poultry flocks and resulting in monetary losses for treatment and control^1^. MG has both horizontal and vertical transmission. MG infection can cause a high feed conversion ratio, egg production loss, poor hatchability, and carcass degradation^1^. Stipkovits and Kempf^2^ investigated the economic loss from MG and found a 10–20% drop in egg production in infected layers and a body weight loss of 10–20% in infected broilers. In Thailand, approximately 25% of all laying hens in the poultry industry are infected with MG, leading to a loss of approximately 15 million U.S. dollars due to a decrease in egg production^3^.

Due to this widespread MG infection, vaccination is an important preventive strategy generally used in the Thailand poultry industry. Live vaccine strains, including the F, ts-11, and 6/85 strains, and inactivated MG vaccines have been used for years^1^. In particular, the F strain is one of the most effective vaccine strains and is widely used in Thailand. Therefore, a technique to differentiate between vaccine and field MG strains in flocks with suspected MG infection is needed. Several studies have investigated techniques for MG classification^4–9^. For example, gene targeted sequencing (GTS) analysis was developed by Ferguson et al.^4^ This technique has been used to determine the gene sequences of partial surface proteins of MG, including the *gap*A, *mgc*2, *pvp*A and MGA_0319 genes. The multilocus sequence typing scheme (MLST) is a technique that many studies have used and is regarded as the gold standard for bacterial typing^6,10,11^. This technique uses MG housekeeping genes for molecular identification, which is an effective way to determine the relationship between MG strains. Both GTS and MLST have been widely used to monitor and characterize MG strains^6^. Additionally, the whole genome sequence (WGS) can be used to analyse the entire genomic sequence of MG^12,13^. High-resolution melt (HRM) curve analysis is another new molecular technique that classifies MG strains by using the *vlh*A, *pvp*A, *gap*A, and *mgc*2 genes as well as the 16S-23S rRNA intergenic space region (IGSR) with conventional and real-time PCR^8,9^. The most commonly used technique in Thailand is random amplification of polymorphic DNA (RAPD). However, RAPD has low reproducibility, and results from different laboratories cannot be compared^14,15^. Sequencing is a potential technique for MG classification. MG strains can be differentiated with partial DNA sequences and compared among laboratories in different areas or countries^4,16,17^. In addition, gene targeted sequencing (GTS) is a cost-efficient and affordable method for use in developing countries, including Thailand, where advanced techniques are not generally feasible.

The important genes of MG, including *gap*A, *mgc*2, *pvp*A and MGA_0319 (*lp*), have been investigated in several epidemiological studies^4,18,19^. In Thailand, Limsatanun et al.^20^ classified MG strains with partial *mgc*2 gene sequences; thus, the partial *mgc*2 gene can be used to classify Thai MG strains from vaccine strains and various strains from different countries. However, partial *mgc*2 gene classification is not a reliable method for MG characterization^4^.

The aim of this study was to determine a GTS technique for differentiating field and vaccine MG strains in commercial chicken flocks from different regions in Thailand. This is the first study to use 4 partial MG gene sequences for commercial MG classification in Thailand.

## Results

### PCR amplification

All twenty-four Thai MG isolates were detected by MG-specific PCR amplification following the Lauerman method^21^. To amplify partial *mgc*2 genes, which were 615 bp in size, 22 Thai MG isolates were successfully amplified and sequenced. According to the specific partial *gap*A PCR with 306 bp, 21 Thai MG isolates were positive and included in the phylogenetic analysis, while 20 samples of Thai MG isolates were successfully amplified using the *pvp*A and MGA_0319 (lp) primers with lengths of 456 and 495 bp, respectively. All nucleotide sequences from Thai MG isolates in this study were submitted to GenBank and given accession numbers (Table 1).

**Table 1:** MG PCR Primers for MG characterization.

Primer	Oligonucleotide sequence (5′-3′)	Reference
*gap*A-3F	TTCTAGCGCTTTAGCCCTAAACCC	Ferguson et al.^4^
*gap*A-3R	CTTGTGGAACAGCAACGTATTCGC	
*pvp*A-3F	GCCAMTCCAACTCAACAAGCTGA	
*pvp*A-3R	GGACGTSGTCCTGGCTGGTTAGC	
*lp* -F	CCAGGCATTTAAAAATCCCAAAGACC	
*lp*-R	GGATCCCATCTCGACCACGAGAAAA	
*mgc*2-1F	GCTTTGTGTTCTCGGGTGCTA	
*mgc*2-1R	CGGTGGAAAACCAGCTCTTG	

### Phylogenetic analysis

**Figure 1:**
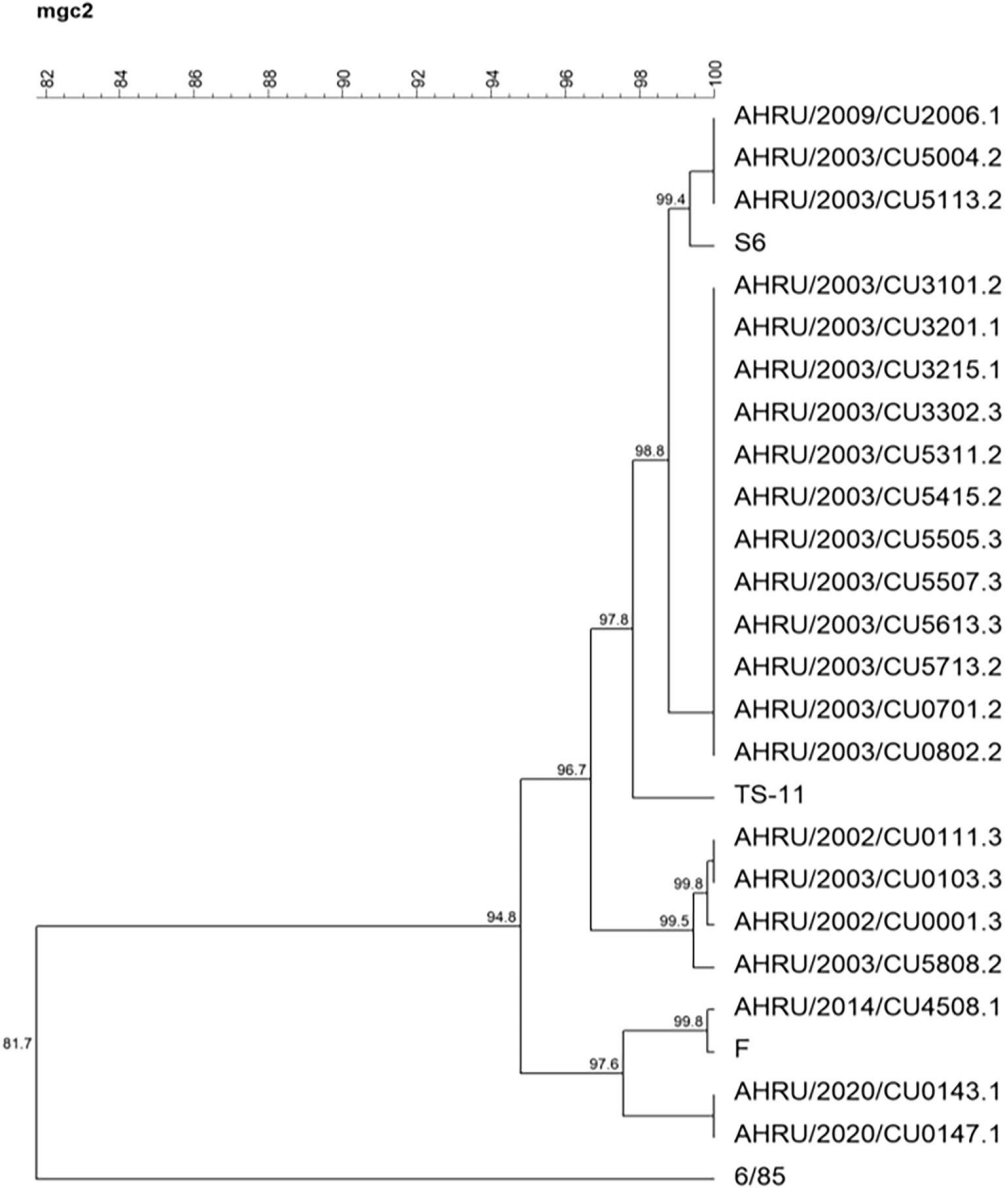
A phylogenetic tree of Thai MG and reference strains based on the alignment of the partial *mgc2* gene was constructed with the unweighted pair group method with arithmetic mean (UPGMA) using Bionumeric version 7.6 software.

**Figure 2:**
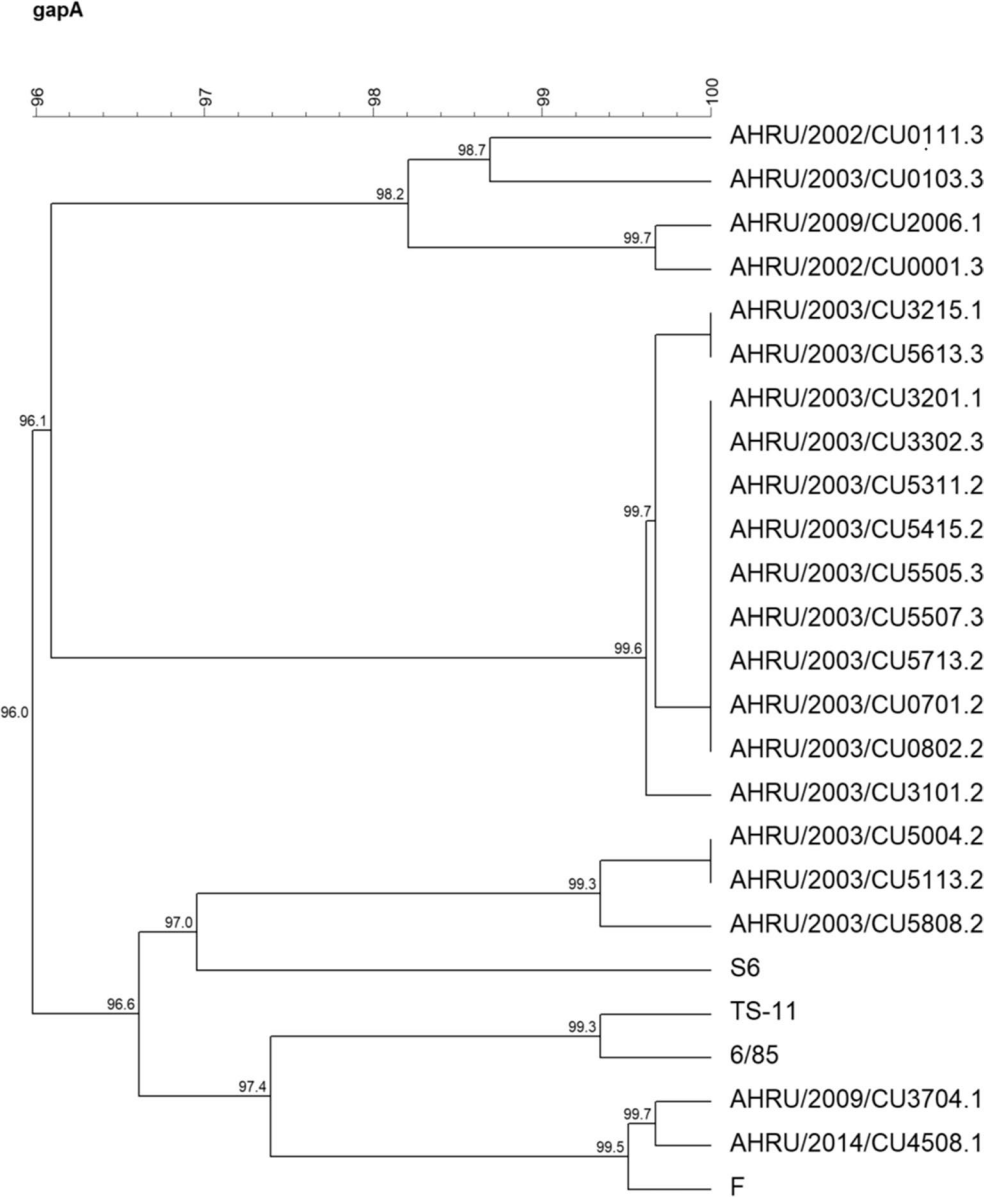
A phylogenetic tree of Thai MG and reference strains based on the alignment of the partial *gapA* gene was constructed with the unweighted pair group method with arithmetic mean (UPGMA) using Bionumeric version 7.6 software.

**Figure 3:**
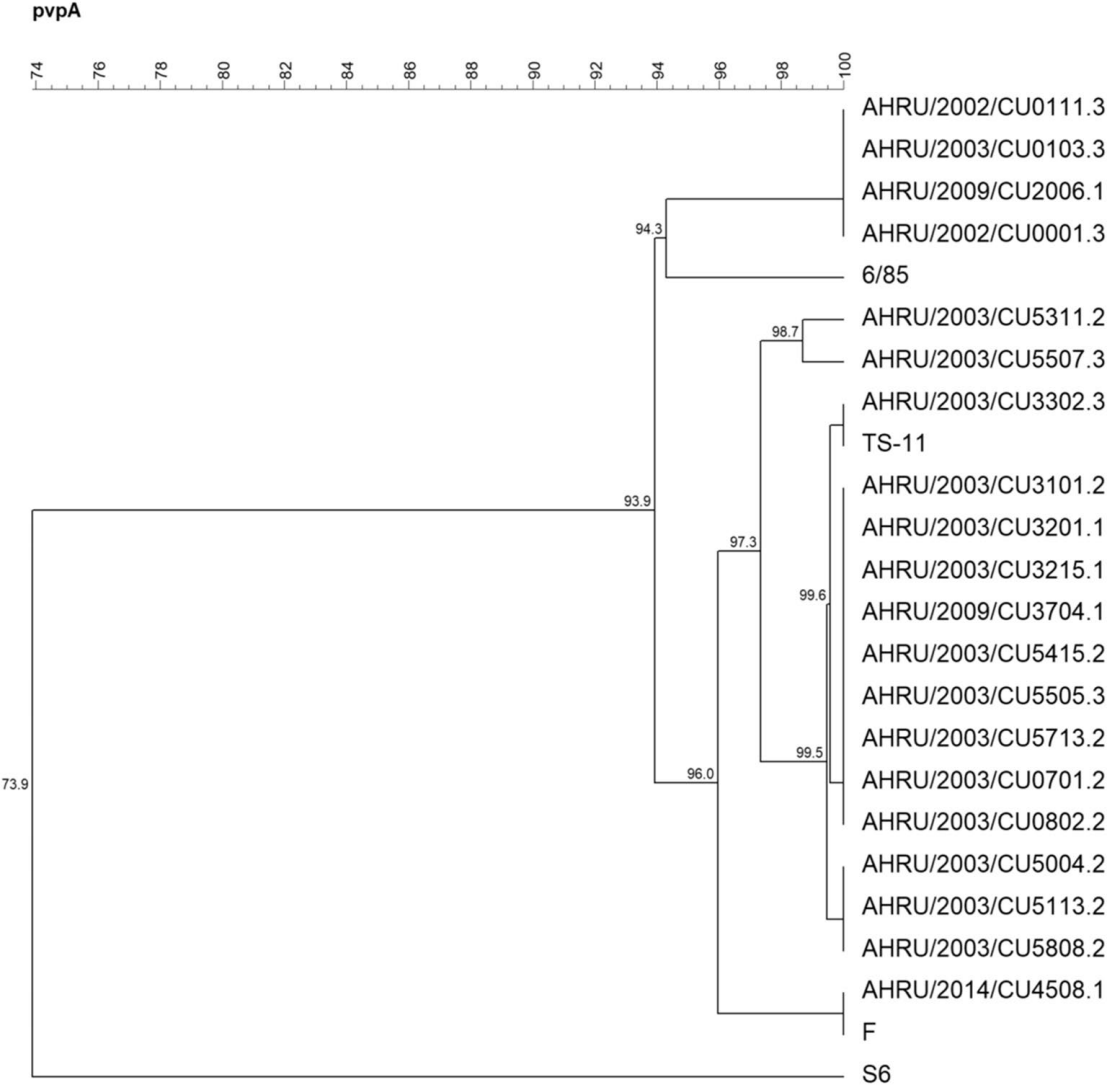
A phylogenetic tree of Thai MG and reference strains based on the alignment of the partial *pvpA* gene was constructed with the unweighted pair group method with arithmetic mean (UPGMA) using Bionumeric version 7.6 software.

**Figure 4:**
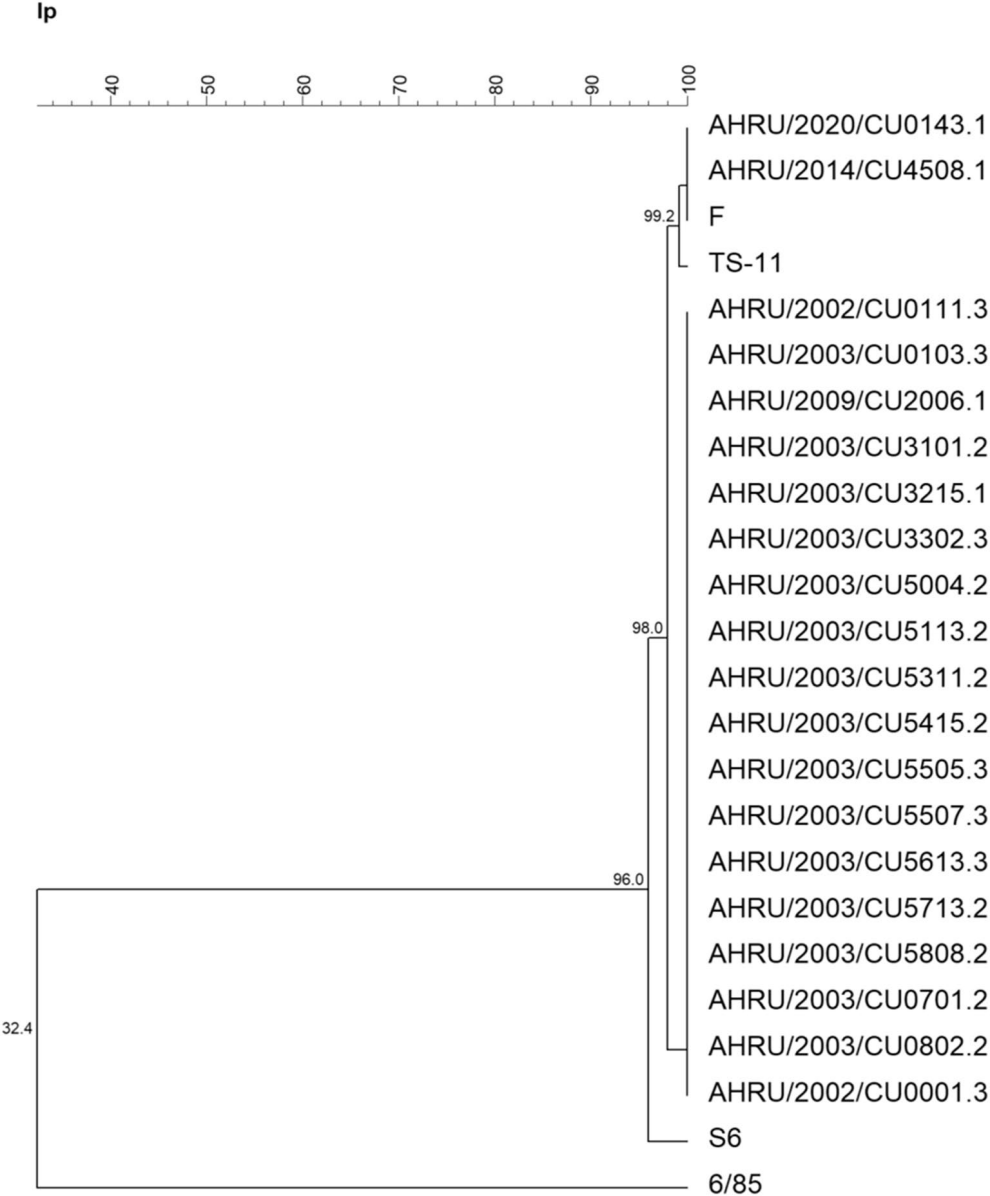
A phylogenetic tree of Thai MG and reference strains based on the alignment of the partial *pvpA* gene was constructed with the unweighted pair group method with arithmetic mean (UPGMA) using Bionumeric version 7.6 software.

The phylogenetic tree based on the partial *mgc*2 gene demonstrated that 3 Thai MG isolates were closely related to the F strain. AHRU/2014/CU4508.1 was grouped together with the F strain, while AHRU/2020/CU0143.1 and AHRU/2020/0147.1 showed 97.6% genetic similarity to the F strain (Fig. 1). According to the phylogenetic tree based on the partial *gapA* gene, AHRU/2014/CU4508.1 showed 99.5% genetic similarity to the F strain. AHRU/2020/CU3704.1 was also grouped with the F strain (Fig. 2). The phylogenetic analysis of the partial *pvp*A gene placed all Thai MG isolates in the same cluster except the reference strain S6 (Fig. 3). Four Thai MG isolates showed 94.3% genetic similarity to the 6/85 strain. AHRU/2014/CU450 8.1 was grouped with the F strain with 100% similarity. The partial *lp* gene sequences of Thai MG were compared with reference strains. The 6/85 strain was grouped into different Clusters. AHRU/2014/CU4508.1 and AHRU/2020/CU0143.1 had 100% genetic similarity to the F strain and 99.2% genetic similarity to the ts-11 strain (Fig. 4). The genetic similarity of Thai MG strains and F strain is shown in Table 2. The *lp* gene showed the highest similarity of genetic sequences (98.2–100%) between the F strain and Thai MG strains. The phylogenetic trees with DNA sequence data are available in the Supplementary Information.

**Table 2:** The genetic similarity (%) between the F strain and Thai MG isolates (Estimated from the number of base substitutions using the maximum composite likelihood model).

Strain	F strain			
	*mgc*2	*gap*A	*pvp*A	lp gene
AHRU/2002/CU0001.3	94.7	98.6	94.8	98.2
AHRU/2002/CU0111.3	94.6	98.1	94.8	98.2
AHRU/2003/CU0103.3	94.6	98.1	94.8	98.2
AHRU/2003/CU0701.2	93.4	98.0	98.6	98.2
AHRU/2003/CU0802.2	93.4	98.0	98.6	98.2
AHRU/2003/CU3101.2	93.4	98.1	98.6	98.2
AHRU/2003/CU3201.1	93.4	98.0	98.6	ND
AHRU/2003/CU3215.1	93.4	97.8	98.6	98.2
AHRU/2003/CU3302.3	93.4	98.0	98.6	98.2
AHRU/2003/CU5004.2	93.7	98.4	98.2	98.2
AHRU/2003/CU5113.2	93.7	98.4	98.2	98.2
AHRU/2003/CU5311.2	93.4	98.0	92.1	98.2
AHRU/2003/CU5415.2	93.4	98.0	98.6	98.2
AHRU/2003/CU5505.3	93.4	98.0	98.6	98.2
AHRU/2003/CU5507.3	93.4	98.0	95.2	98.2
AHRU/2003/CU5613.3	93.4	97.8	ND	98.2
AHRU/2003/CU5713.2	93.4	98.0	98.6	98.2
AHRU/2003/CU5808.2	95.1	98.7	98.2	98.2
AHRU/2009/CU2006.1	93.7	98.7	94.8	98.2
AHRU/2009/CU3704.1	ND	99.5	98.6	ND
AHRU/2014/CU4508.1	99.8	99.5	100.0	100.0
AHRU/2020/CU0143.1	97.6	ND	ND	100.0
AHRU/2020/CU0147.1	97.6	ND	ND	ND

*ND* not detected.

## Discussion

Avian mycoplasmosis is an important disease-causing pathogen in the poultry industry with substantial economic impacts. Live, inactivated, and recombinant MG vaccines have been used in Thailand for a long time. Due to the increased use of MG vaccines, differentiation between field and vaccine strains is needed. Molecular characterization of MG has been investigated in many countries^4,5,7,22,23^. This study is the first to use the GTS technique on Thai MG strains with 4 partial MG gene sequences. The partial *mgc* gene has been used for MG characterization in many epidemiological studies^6,20,24^. It encodes the MGC2 protein, which coordinates with the *gapA* gene-encoded protein for cell attachment^25^ and is involved in MG immunogenicity^12,26^.

In a previous study, Armour et al.^22^ investigated MG isolates from South Africa using intergenic spacer regions (IGSRs), *mgc2* and *gapA* genes. Thirty-six MG isolates were classified into 8 types by the *mgc2* gene and 2 types by the *gapA* gene. Thus, the *mgc2* gene had a higher discriminatory power than the *gapA* gene. Another study in Russia conducted an epidemiological investigation of MG^7^. The results showed that *mgc2* gene had good discriminatory power, while gapA did not provide a good discriminatory index for MG classification. However, the use of only a single gene for classification could not determine the similarity between MG isolates. Additionally, some MG isolates were negative for the *mgc2* gene according to the PCR assay, resulting in a failure to obtain *mgc2* sequences^5,6,19,22^; thus, using only one partial gene sequence is insufficient to characterize MG.

In the present study, the *lp* gene of Thai MG isolates was more conserved than the *gapA, mgc2* and *pvpA* genes, as 18 out of 20 Thai MG isolates showed 100% genetic similarity on this gene. The use of partial *gapA* showed the highest genetic variation among Thai isolates. These results contradicted those of previous studies^4,7,22^, which indicated that MG isolates from the same area would have lower genetic diversity than MG isolates from different regions^22^. In the present study, Thai MG isolates were identified with 4 genes using the phylogenetic tree (UPMGA) method. AHRU/2014/CU4508.1 had the closest genetic relationship to the F strain. The UPMGA results showed that AHRU/2014/CU4508.1 was grouped with the F strain on all 4 partial gene analyses. Interestingly, all Thai MG isolates in this study were collected from farms with unvaccinated flocks. In Thailand, poultry breeders and layers are widely vaccinated with the F strain. Interestingly, the AHRU/2014/CU4508.1 isolate from these farms might have been contaminated from other farms with vaccinated flocks. The F strain from the live MG vaccine can be transmitted both horizontally and vertically^27–29^. Furthermore, several epidemiological studies have shown that the F strain can cause MG outbreaks if it spreads from vaccinated to nonvaccinated flocks^5,24,30^. Other Thai MG isolates in this study varied in genetic classification depending on the gene analysed. The results of *gapA* and *mgc2* gene analysis showed that AHRU/2003/CU5113.2 and AHRU/2003/CU5808.2 were grouped with the S6 strain with 97% and 99.4% genetic similarity, respectively. In contrast, using the partial *pvpA* gene sequence indicated that the S6 strain was separated from all Thai MG isolates, including AHRU/2003/CU5113.2 and AHRU/2003/CU5808.2. These results indicated that AHRU/2003/CU5113.2 and AHRU/2003/CU5808.2 might be genetically related to the S6 strain. DNA sequences of all 4 virulence genes could not be obtained for some Thai MG isolates. For example, the Thai MG isolate AHRU/2009/CU3704 could only be classified by phylogenetic analysis of *gapA* and *pvpA* genes because it was negative for *mgc*2 and *pvpA* according to the PCR analysis. Plausibly, this lack of detection could be because of the poor quality of DNA due to the presence of multiple strains in the broth medium sample and/or genetic mutations between and within MG strains^6,31,32^.

In conclusion, the Thai MG isolates in this study could be differentiated with partial MG genes, including the *gap*A, *mgc*2, *pvp*A and MGA_0319 (*lp*) genes. All Thai MG isolates could be classified with at least 2 out of 4 partial gene sequences, especially the partial *gap*A and *mgc*2 genes, which had satisfactory discriminatory power for Thai MG characterization. Using partial DNA sequencing for MG characterization is an effective and reproducible method for establishing the genetic relationship between MG strains and differentiating between vaccine and field strains. In addition, this study was the first epidemiological study of Thai MG strains to use 4 partial MG gene sequences, demonstrating the genetic diversity of circulating MG strains in Thailand. In future studies, the GTS technique should be implemented along with other molecular techniques, including a multilocus sequence typing scheme, to provide more epidemiological and evolutionary data and improve the system for monitoring MG outbreaks in poultry farms in Thailand.

## Materials and methods

### MG isolates

Twenty-four Thai MG isolates were used in this study. All isolates were collected during 2003–2020 by Prof. Somsak Pakpinyo, Department of Veterinary Medicine, Faculty of Veterinary Science, Chulalongkorn University. All MG isolates were collected from choanal cleft of dead chicken and were propagated in FMS medium supplemented with 15% swine serum following previously reported methods^33^ and incubated at 37 °C until the broth colour changed from pink to orange. All isolates were confirmed as MG by polymerase chain reaction (PCR) assay^21^.

### Molecular typing

The DNA from each Thai MG isolate was extracted with an equal volume of phosphate buffered saline (PBS) and then amplified by PCR. The primers in this study were designed by Ferguson^4^ (Table 3). A PCR assay was performed to detect the partial *gap*A, *pvp*A, MGA_0319 and *mgc*2 genes. The PCR mixture consisted of 500 mM KCl, 100 mM Tris–HCl (pH 8.3), 1.25 mM MgCl2, 1 mM dNTP (Thermo Scientific, Vilnius, Lithuania), 10 pmol each of primer (Qiagen®, Valencia, CA, USA), 1.25 µl of Taq polymerase (Promega, Madison, WI, USA) and 2.5 µl (125 ng) of the DNA template. The amplification reaction was performed in a DNA thermal cycler at 94 °C for 3 min, followed by 40 cycles of 94 °C for 20 s, 55–60 °C for 40 s, 72 °C for 60 s, and 72 °C for 5 min for the *gap*A, *pvp*A, MGA_0319 (*lp*) and *mgc*2 genes. The PCR products were 332, 702, 590 and 824 bp, respectively^4^.

**Table 3:** Thai MG strains and GenBank accession numbers.

Strain	Type of chicken	Source	Accession number			
			*mgc*2	*gap*A	*pvp*A	lp gene
F	–	Vaccine	MW617973	MW617946	MW617933	MW617913
S6	–	Laboratory	MW617972	MW617947	MW617934	MW617934
TS-11	–	Vaccine	MW617971	MW617954	MW617945	MW617945
6/85	–	Vaccine	MW617974	MW617966	MW617930	MW617930
AHRU/2002/CU0001.3	Breeder	Central region	MW617980	MW617957	MW617943	MW617919
AHRU/2002/CU0111.3	Breeder	Central region	KX268616	MW617959	MW617944	MW617920
AHRU/2003/CU0103.3	Breeder	Central region	KX268617	MW617958	MW617940	MW617918
AHRU/2003/CU0701.2	Breeder	Eastern region	KX268618	MW617948	MW617931	MW617908
AHRU/2003/CU0802.2	Breeder	Eastern region	KX268619	MW617949	MW617935	MW617909
AHRU/2003/CU3101.2	Breeder	Eastern region	KX268620	MW617969	MW617929	MW617917
AHRU/2003/CU3201.1	Breeder	Eastern region	MW617977	MW617960	MW617926	–
AHRU/2003/CU3215.1	Breeder	Eastern region	KX268621	MW617970	MW617925	MW617904
AHRU/2003/CU3302.3	Breeder	Eastern region	KX268622	MW617965	MW617924	MW617916
AHRU/2003/CU5004.2	Layer	Central region	KX268624	MW617955	MW617927	MW617905
AHRU/2003/CU5113.2	Layer	Central region	KX268625	MW617956	MW617923	MW617902
AHRU/2003/CU5311.2	Breeder	Eastern region	KX268626	MW617964	MW617922	MW617903
AHRU/2003/CU5415.2	Breeder	Eastern region	KX268627	MW617950	MW617938	MW617907
AHRU/2003/CU5505.3	Breeder	Eastern region	KX268628	MW617963	MW617936	MW617901
AHRU/2003/CU5507.3	Breeder	Eastern region	KX268629	MW617962	MW617937	MW617900
AHRU/2003/CU5613.3	Layer	Western region	MW617975	MW617961	–	MW617899
AHRU/2003/CU5713.2	Layer	Eastern region	KX268630	MW617951	MW617939	MW617910
AHRU/2003/CU5808.2	Layer	Central region	KX268631	MW617952	MW617941	MW617911
AHRU/2009/CU2006.1	Layer	Western region	KX268632	MW617953	MW617942	MW617912
AHRU/2009/CU3704.1	Breeder	Western region	–	MW617968	MW617932	–
AHRU/2014/CU4508.1	Breeder	Western region	MW617976	MW617967	MW617928	MW617906
AHRU/2020/CU0143.1	Breeder	Central region	MW617979	–	–	MW617915
AHRU/2020/CU0147.1	Breeder	Central region	MW617978	–	–	–

### Reference sequences

Four reference strains were used in this study. The F strain was the vaccine strain provided by a local distributor (MSD, Thailand). The S6 strain was obtained from ATCC (15302). The ts-11 and 6/85 strain sequences were obtained from Prof. Somsak Pakpinyo, Department of Veterinary Medicine, Faculty of Veterinary Science, Chulalongkorn University.

### DNA sequence analysis

Amplified PCR products of MG-targeted gene-positive extracts were submitted to determine the sequence. Partial *mgc2* gene sequences (Accession Numbers KX268616–KX268632) from 16 Thai MG isolates had been submitted to GenBank in a previous study^20^ (Table 3.) All sequences were analysed with the Editseq program (Lasergene, DNASTAR Inc., USA), and a consensus was constructed with the Seqman program (Lasergene, DNASTAR Inc., USA). Thai MG isolates and reference gene sequence data were aligned to construct a phylogenetic tree in Bionumeric version 7.6 software (Applied Maths, Sint-Martens-Latem, Belgium). Cluster analysis was performed with the UPGMA method. The similarity coefficients of Thai MG isolates and reference strains were determined from multiple sequence alignments.

## Supplementary Information

Supplementary Information.

## Data Availability

All data generated or analysed during this study are included in this published article [and its Supplementary Information files]. The sequence data are available at the NCBI Nucleotide (https://www.ncbi.nlm.nih.gov/nuccore); see Table 3 for sample accession numbers.
